# Immunization with gingipain A hemagglutinin domain of *Porphyromonas gingivalis* induces IgM antibodies binding to malondialdehyde-acetaldehyde modified low-density lipoprotein

**DOI:** 10.1371/journal.pone.0191216

**Published:** 2018-01-12

**Authors:** Mikael Kyrklund, Outi Kummu, Jari Kankaanpää, Ramin Akhi, Antti Nissinen, S. Pauliina Turunen, Pirkko Pussinen, Chunguang Wang, Sohvi Hörkkö

**Affiliations:** 1 Medical Microbiology and Immunology, Research Unit of Biomedicine, Faculty of Medicine, University of Oulu, Oulu, Finland; 2 Medical Research Center and Nordlab Oulu, University Hospital and University of Oulu, Oulu, Finland; 3 Oral and Maxillofacial Diseases, University of Helsinki and Helsinki University Hospital, Helsinki, Finland; University of Leicester, UNITED KINGDOM

## Abstract

Treatment of periodontitis has beneficial effects on systemic inflammation markers that relate to progression of atherosclerosis. We aimed to investigate whether immunization with A hemagglutinin domain (Rgp44) of *Porphyromonas gingivalis (Pg)*, a major etiologic agent of periodontitis, would lead to an antibody response cross-reacting with oxidized low-density lipoprotein (OxLDL) and how it would affect the progression of atherosclerosis in low-density lipoprotein receptor-deficient (LDLR^-/-^) mice. The data revealed a prominent IgM but not IgG response to malondialdehyde-acetaldehyde modified LDL (MAA-LDL) after Rgp44 and *Pg* immunizations, implying that Rgp44/*Pg* and MAA adducts may share cross-reactive epitopes that prompt IgM antibody production and consequently confer atheroprotection. A significant negative association was observed between atherosclerotic lesion and plasma IgA to Rgp44 in Rgp44 immunized mice, supporting further the anti-atherogenic effect of Rgp44 immunization. Plasma IgA levels to Rgp44 and to *Pg* in both Rgp44- and *Pg*-immunized mice were significantly higher than those in saline control, suggesting that IgA to Rgp44 could be a surrogate marker of immunization in *Pg*-immunized mice. Distinct antibody responses in plasma IgA levels to MAA-LDL, to *Pg* lipopolysaccharides (*Pg*-LPS), and to phosphocholine (PCho) were observed after Rgp44 and *Pg* immunizations, indicating that different immunogenic components between Rpg44 and *Pg* may behave differently in regard of their roles in the development of atherosclerosis. Immunization with Rgp44 also displayed atheroprotective features in modulation of plaque size through association with plasma levels of IL-1α whereas whole *Pg* bacteria achieved through regulation of anti-inflammatory cytokine levels of IL-5 and IL-10. The present study may contribute to refining therapeutic approaches aiming to modulate immune responses and inflammatory/anti-inflammatory processes in atherosclerosis.

## Introduction

Atherosclerosis plays a major role in cardiovascular disease (CVD) and is the leading cause of death worldwide [1]. It is considered a chronic inflammatory disease [2] whose recognized risk factors include environmental, genetic, and inflammatory elements [1]. Low-density lipoprotein (LDL) particles circulate in the blood and can accumulate in the arterial intima leading to the formation of arterial plaques [3]. Oxidative modification of LDL, which creates oxidized LDL particles (OxLDL) and formation of malondialdehyde-modified LDL (MDA-LDL), is the first step in the development of atherosclerosis. MDA is unstable and can further react with acetaldehyde to form malondialdehyde acetaldehyde-modified LDL (MAA-LDL) [4–6]. MAA adduct has been proposed to be one of the main epitopes for the immune system after MDA modification of proteins or lipids in atherosclerosis [4,5,7]. Phosphocholine (PCho) is another common epitope of oxidized phospholipids in OxLDL. It is also exposed on some microorganisms, e.g. *Streptococcus pneumoniae*, and on apoptotic cells. A well characterized natural IgM antibody, T15/EO6, binds both microbial antigens and stress-induced self-antigens, such as OxLDL, via the recognition of PCho [8]. Active immunization with PCho and passive immunization with anti-PCho ameliorate the development of atherosclerosis in apolipoprotein E-null mice [9].

Periodontitis is known to associate with an increased risk of atherosclerosis. It is a chronic inflammatory disease affecting tooth-supporting tissue, and a mild form of the disease is present in the majority of adult population. Treatment of periodontitis improves systemic inflammation markers that also relate to development of atherosclerosis [10,11]. *Porphyromonas gingivalis (Pg)* is one of the most important bacterial species in chronic periodontitis [12]. *Pg* can be detected in arterial plaques [13] and can also invade endothelial cells in human coronary arteries [14]. *Pg* lipopolysaccharide (*Pg*-LPS) is the major component of the outer membrane of the bacteria that stimulates the innate immune system and is a key virulence factor that has been shown to induce production of cytokines [15]. LPS works through Toll-like receptor 4 (TLR4). *Pg* has also been show to act through TLR2 engagement, and TLR2-mediated effects are reported to be important in alveolar bone loss in periodontal disease and atherosclerosis [16,17]. Although the exact mechanisms linking periodontitis to atherosclerosis are still not defined, the association between the two conditions is partially explained by previous studies [10].

Compelling evidence links inflammation and immune response to all phases of atherosclerotic lesion development [18]. It has been suggested that IgM autoantibodies are atheroprotective whereas IgG autoantibodies are much more heterogeneous, but in general, they directly correlate with CVD manifestations in univariate analyses [5,18–20]. Little information is available about the role of IgA in atherosclerosis. There appears to be an association between high serum IgA titers and advanced vascular disease and myocardial infarction [18].

A mouse monoclonal IgM antibody (MDmAb) against MDA-LDL has previously been cloned in our lab [21]. The antibody recognized gingipain protease produced by *Pg* and bound to recombinant gingipain including a hemagglutinin/adhesin domain (Rgp44 domain, amino acids 717–1135) [21]. We have also previously immunized mice with *Pg* bacteria, which led to production of IgM antibodies to MDA-LDL and diminished atherosclerosis [22]. In this study, we aimed to investigate whether immunization with gingipain A hemagglutinin domain (Rgp44) would induce antibody responses to malondialdehyde acetaldehyde (MAA) adducts in mice. Plasma antibodies to OxLDL, aortic atherosclerotic lesions, and inflammatory/anti-inflammatory cytokines were examined and the association among these biomarkers was explored.

## Materials and methods

### Mice immunization and diets

The study was approved by the National Animal Experimental Board of Finland (ESAVI/6097/04.10.03/2011). Forty female LDLR^-/-^ mice (about 8 week-old) were randomly divided into three immunization groups: adhesin/hemagglutinin domain of RgpA of *Porphyromonas gingivalis* (Rgp44, n = 13), *Porphyromonas gingivalis* (*Pg*, n = 13), and phosphate buffered saline (Saline, n = 14). The length of the study was 40 weeks, and procedures were carried out as shown in S1 Fig. Mice were first injected subcutaneously with 50 μg protein, followed by intraperitoneal booster injections of 25 μg of protein every 2 weeks for five boosters and every month for six boosters. No adjuvants were used in the study. The mice were on chow diet (4.4% fat and 0.2% cholesterol) for 13 weeks, followed by high-fat diet (HFD; 21.2% fat and 0.2% cholesterol, Western Diet TD88137 Harlan Tekland) for 27 weeks. Blood samples were taken from the hind leg before immunizations and before HFD, and from the *vena cava* at the time of sacrifice. Mouse aortas were collected for *en face* analysis after sacrifice.

### Bacteria

*Porphyromonas gingivalis* (*Pg*), serotype ATCC 33277, was cultured on Brucella agar plates supplemented with 5% horse blood, 100 mg/mL vitamin K1, and 5 μg/mL hemin anaerobically at 37°C for 6 days. Culture purity was determined by gram-staining and colony morphology. Heat-inactivated *Pg* was made by incubation of bacteria suspension in PBS at 60°C for 1 h and frozen at -80°C in small aliquots until further use.

### Production of recombinant Rgp44 protein

*Escherichia coli BL21(DE3)* was used to express recombinant protein of *Pg* arginine-specific gingipain A adhesion/hemagglutinin domain (Rgp44). The polymerase chain reaction amplification and ligation of Rgp44 to pET32 Xa LIC vector were performed as described previously [21]. ProBond Purification protocols were used with slight modification for the production of histidine-tagged recombinant protein (Invitrogen, Carlsbad, CA, USA). *E*.*coli* cells were harvested by centrifugation at 4,500 × g for 15 min and native lysis buffer (50 mM Tris, 25 mM sodium chloride, 2.5 mM magnesium chloride, and 0.1 mM calcium chloride, pH 8.0) was used to suspend the cell pellets. Lysozyme was added to 1 mg/mL and the sample was incubated at 30°C for 50 min. Sonication was performed at 50% amplitude for 4 × 10 sec on ice. DNase I (4 U/mL, Fermentas) was added and the sample was incubated at 37°C for 15 min. Cellular debris was collected by centrifugation at 4,500 × g for 15 min. Rgp44 recombinant protein was mainly expressed in insoluble form and accumulated in the inclusion bodies. Rgp44 was purified from *E*.*coli* inclusion bodies by adding denaturing lysis buffer (6 M guadinium hydrochloride in native lysis buffer) for 10 min. The cell lysate was homogenized and agitated gently for 20 min at +4°C. The lysate was centrifuged at 100,000 × g for 30 min at +4°C. Soluble proteins in the supernatant were bound to nickel chelation slurry. Protein purification was done according to the ProBond Purification protocol. Purity of the histidine-tagged Rgp44 recombinant protein was analyzed by sodium dodecyl sulfate polyacrylamide gel electrophoresis (SDS-PAGE).

### LDL isolation and modifications

Low-density lipoprotein (LDL) fraction (density 1.019–1.063 g/mL) was isolated from mouse plasma by sequential density gradient centrifugation [23]. Malondialdehyde acetaldehyde-modified LDL (MAA-LDL) and copper-oxidized LDL (CuOx-LDL) were prepared as described [21]. The extent of lipoprotein modification in each sample was verified by fluorescence measurements (excitation 355 nm, emission 460 nm) and colorimetric measurements with 2,4,6-trinitrobenzene sulfonic acid [24].

### Chemiluminescence immunoassay

The amount of antibodies was determined by chemiluminescence immunoassay as described elsewhere [21]. Rgp44, heat-inactivated *Pg*, MAA-LDL, CuOx-LDL, phosphocholine-conjugated bovine serum albumin (PCho-BSA, Biosearch Technologies, Novato, CA, USA), BSA, *Pg*-LPS (Invivogen, San Diego, CA, USA) were used as antigens. The antigens were coated (5 μg/mL) in phosphate-buffered saline (PBS) with 0.27 mmol/L ethylenediaminetetraacetic acid (EDTA) at 4°C overnight onto white MicroFluor plates (Thermo Scientific, Rockford, IL, USA). The plates were washed with PBS-EDTA three times using an automated plate washer and blocked with PBS-EDTA containing 0.5% fish gelatin at room temperature for 1 h. Plasma samples were diluted and measured in triplicate. Antibody binding was detected with an appropriate alkaline phosphatase-labeled goat anti-mouse secondary antibody [Sigma-Aldrich, goat anti-mouse IgM (μ-chain specific)-alkaline phosphatase, goat anti-mouse IgG (Fc specific)-alkaline phosphatase, and goat anti-mouse IgA (α-chain specific)-alkaline phosphatase]. Chemiluminescence was detected using LumiPhos530 substrate (Lumigen Inc. Southfield, MI, USA) with Wallac Victor^3^ multilabel reader, and expressed as relative light units (RLU) measured in 100 milliseconds (ms).

### Determination of mouse atherosclerosis

Mouse atherosclerosis was analyzed using ‘*en face’* analysis of whole aortas. After sacrifice, blood was first collected from *vena cava* and mice were perfused with PBS containing 1 μg/mL butylated hydroxytoluene (BHT) through the left ventricle for 10 min and perfusion-fixed with 10% formalin, 5% sucrose, 20 μmol/L BHT and 3 μmol/L EDTA, pH 7.4 for 10 min. Aortas were cleaned, stained with Sudan IV, and pinned onto paraffin plates. The images were acquired with a Nikon D90 camera and Tokina AT-X 100 PRO D macro lens. Stained aortic plaque areas were analyzed using ImageJ 1.48v analysis software (https://imagej.net/ImageJ) and expressed as percentage of plaque area per total area of aorta.

### Determination of plasma cytokines

Cytokines were measured using mouse Th1/Th2/Th17/Th22 13plex assay (eBioscience, Vienna, Austria). A mixture of coated beads for each analyte to be measured was incubated with 25 μL of undiluted plasma samples or standard mixture. The biotin-conjugated secondary antibody mixture was added to bind to the analytes captured by the first antibodies. Finally, Streptavidin-Phycoerythrin bound to the biotin conjugate leading to the emission of fluorescent signals. The samples were analyzed with FACSCalibur flow cytometer and CellQuest software (BD Biosciences, San Jose, CA) and calculated by FlowCytomix Pro 3.0 Software. In mouse Th1/Th2/Th17/Th22 Flowcytomix 13plex assay the standard range is 27–20,000 pg/mL (IFN-γ, IL-1α, IL-2, IL-4, IL-5, IL-6, IL-10, IL-13, IL-17, IL-21 and TNF-α), 2.7–2,000 pg/mL (IL-22) and 69–50,000 pg/mL (IL-27). The mean intra-assay coefficient of variation of three independent assays with six replicates of four samples is less than 6.4% and the inter-assay coefficient of variation less than 10.9% in all analyses.

### Statistics

Statistical analyses were performed using IBM SPSS statistics version 22.0. Nonparametric Mann-Whitney U test was used to detect differences between the variables in different treatment groups and nonparametric Wilcoxon signed-rank test to analyze differences between the variables within the same treatment groups. The associations were evaluated by Spearman’s correlation coefficient (03C1).

## Results

### Prominent immune responses to Rgp44 and *Pg* bacteria

Female LDLR^-/-^ mice (about 8 weeks old) were randomly divided into three immunization groups (saline, Rgp44, and *Pg*). The experimental timeline is shown in S1 Fig. Recombinant gingipain A hemagglutinin domain (Rgp44) and heat-inactivated whole *Pg* bacteria (*Pg*) induced prominent immune responses in LDLR^-/-^ mice. Plasma IgG and IgM (S2 Fig) antibody levels to both Rgp44 and *Pg* were significantly elevated in Rgp44- and *Pg*-immunized mice when compared to the mice immunized with phosphate buffered saline (saline).

### Plasma IgM antibody levels to MAA-LDL, CuOx-LDL, and PCho increased significantly after Rgp44 and *Pg* immunizations

**Fig 1:**
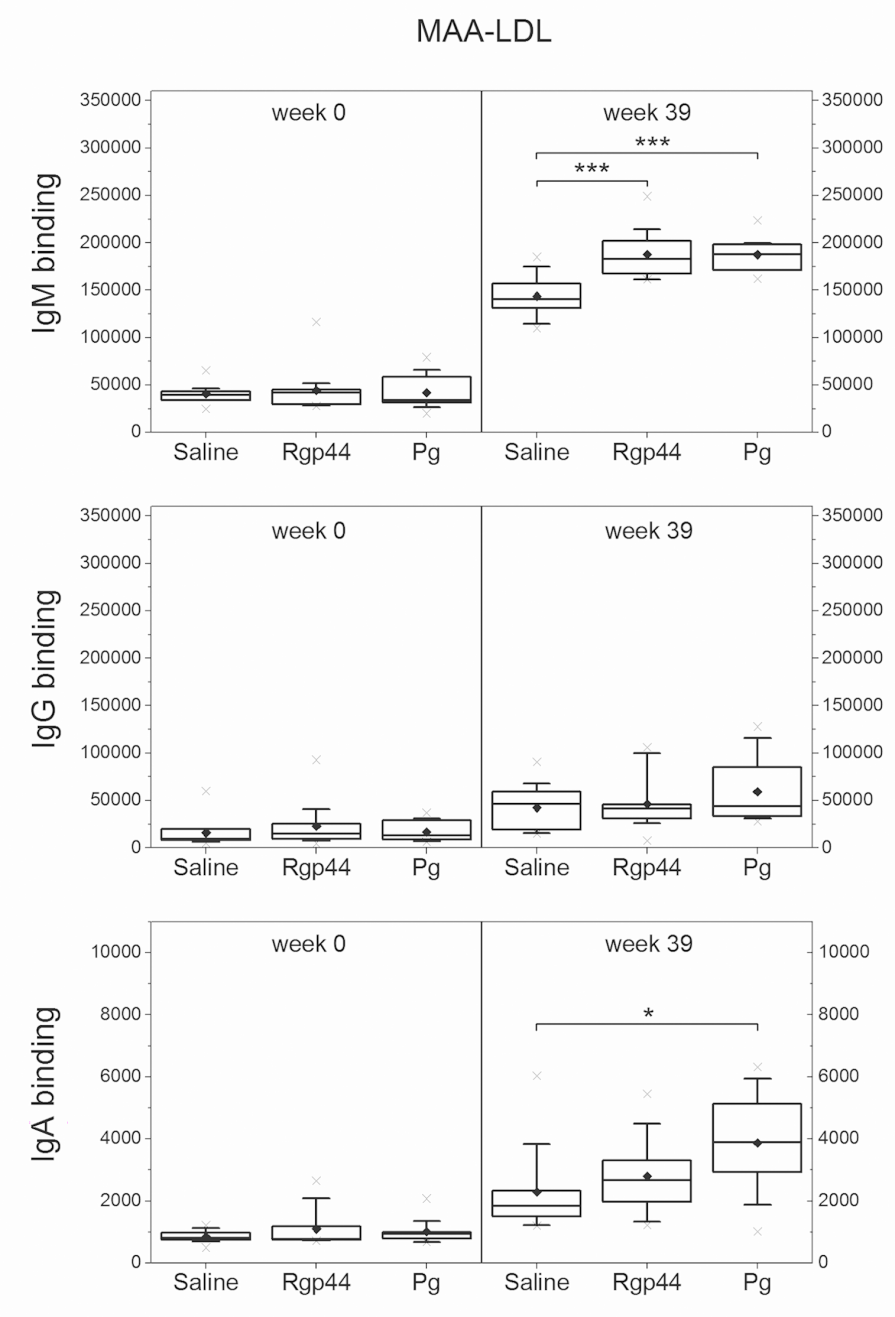
Mouse plasma antibody levels to MAA-LDL before immunization (week 0) and at the end of the study (week 39) in LDLR^-/-^ mice immunized with saline, Rgp44, and heat-inactivated *Pg*. Each mouse plasma sample was measured in triplicate using chemiluminescence immunoassay. The antibody binding is expressed as mean ± SD in relative light units measured in 100 milliseconds (RLU/100ms). Plasma antibody levels as box-whisker plots represent 25%, 50% and 75% of the distribution, where the whiskers represent 10% and 90% distribution of the values and the cross represents the maximum and minimum range. The solid diamonds are the mean values. P-values less than 0.05 are regarded as statistically significant (nonparametric Mann-Whitney U test). * p < 0.05, ** p < 0.01, *** p < 0.001.

**Fig 2:**
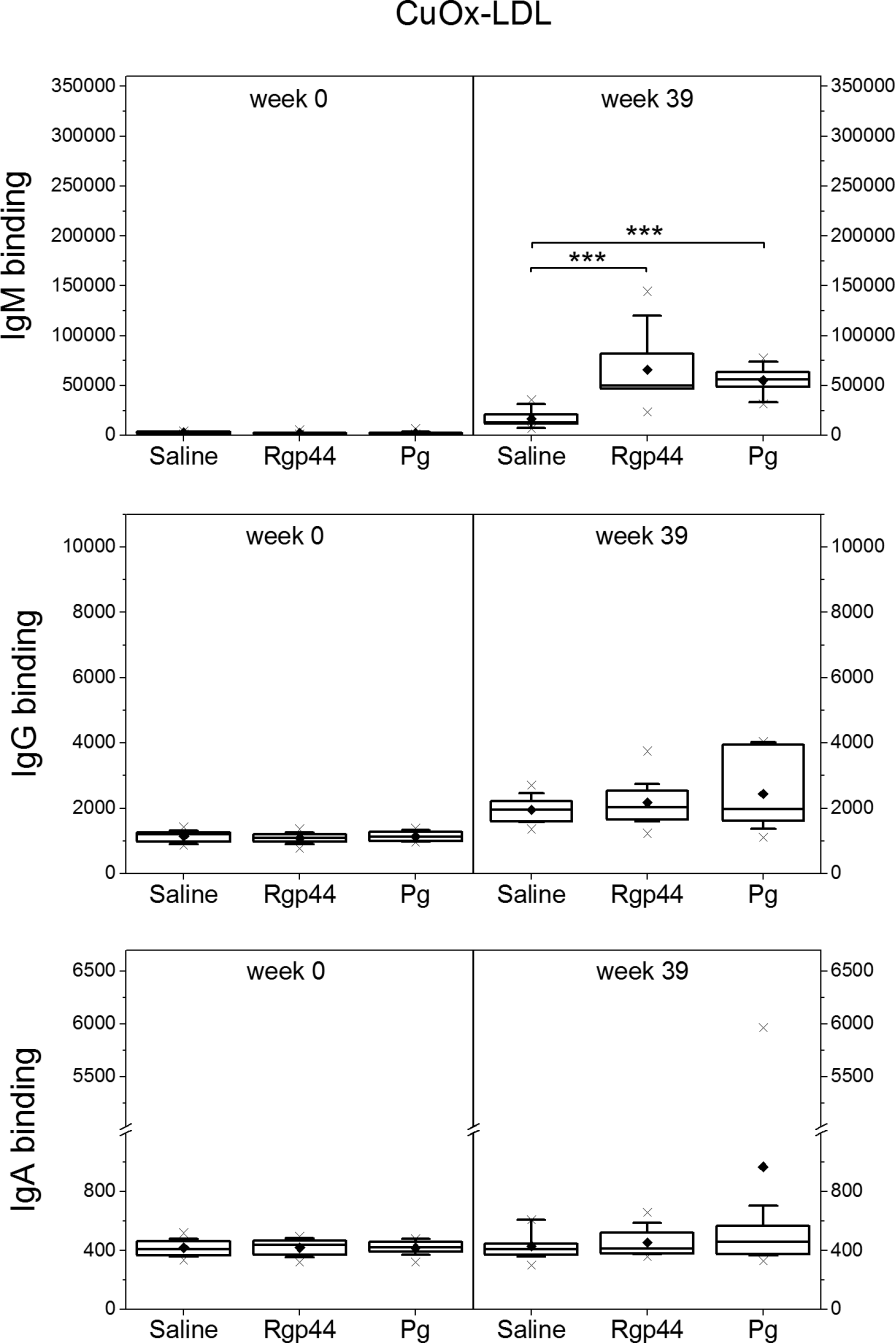
Mouse plasma antibody levels to CuOx-LDL before immunization (week 0) and at the end of the study (week 39) in LDLR^-/-^ mice immunized with saline, Rgp44, and heat-inactivated *Pg*. Each mouse plasma sample was measured in triplicate using chemiluminescence immunoassay. The antibody binding is expressed as mean ± SD in relative light units measured in 100 milliseconds (RLU/100ms). Plasma antibody levels as box-whisker plots represent 25%, 50% and 75% of the distribution, where the whiskers represent 10% and 90% distribution of the values and the cross represents the maximum and minimum range. The solid diamonds are the mean values. P-values less than 0.05 are regarded as statistically significant (nonparametric Mann-Whitney U test). *** p < 0.001.

**Fig 3:**
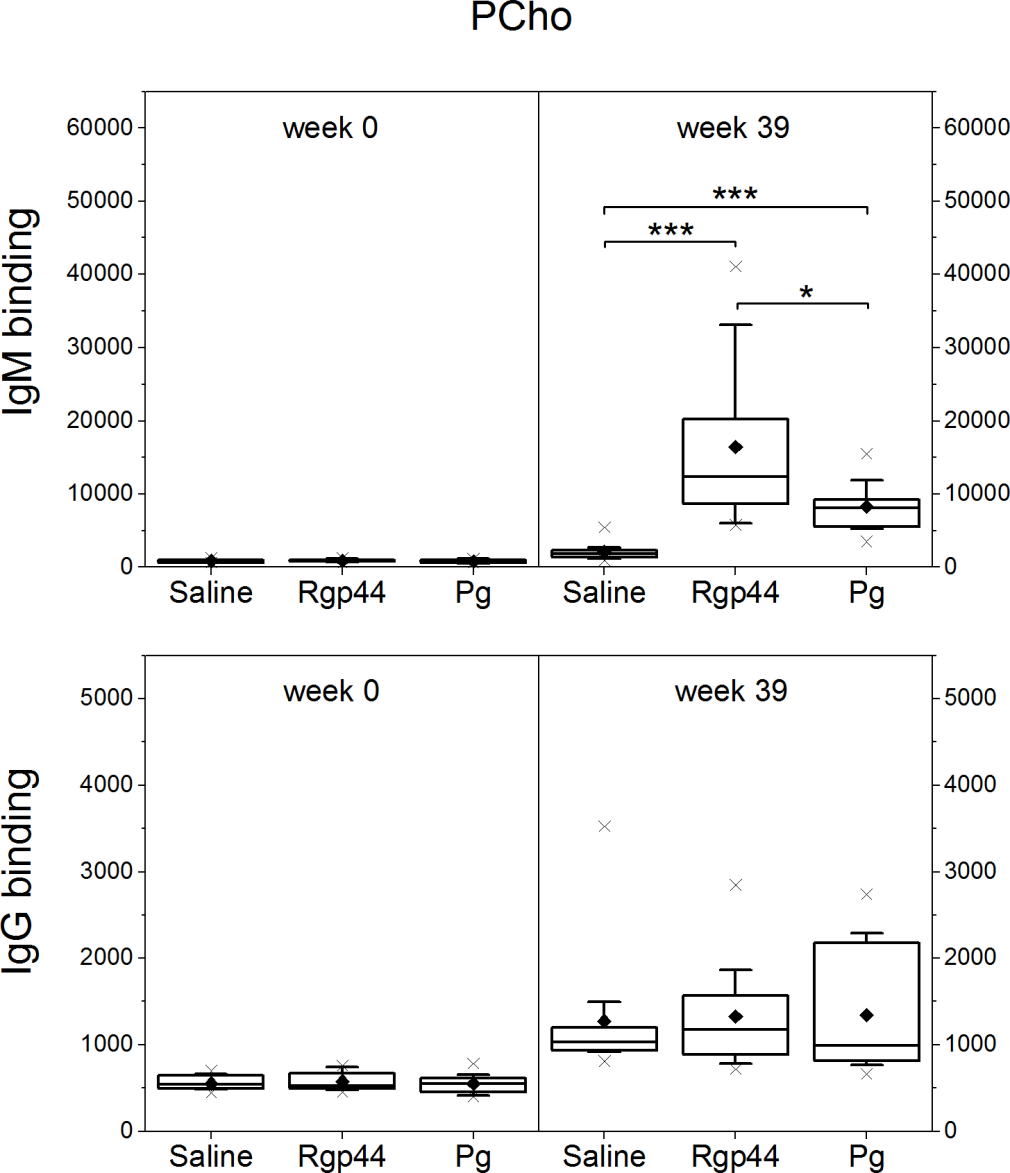
Mouse plasma antibody levels to PCho before immunization (week 0) and at the end of the study (week 39) in LDLR^-/-^ mice immunized with saline, Rgp44, and heat-inactivated *Pg*. Each mouse plasma sample was measured in triplicate using chemiluminescence immunoassay. The antibody binding is expressed as mean ± SD in relative light units measured in 100 milliseconds (RLU/100ms). Plasma antibody levels as box-whisker plots represent 25%, 50% and 75% of the distribution, where the whiskers represent 10% and 90% distribution of the values and the cross represents the maximum and minimum range. The solid diamonds are the mean values. P-values less than 0.05 are regarded as statistically significant (nonparametric Mann-Whitney U test). * p < 0.05, *** p < 0.001.

To investigate whether the immunizations induced the antibodies binding to oxidation-specific epitopes, plasma antibody levels against MAA-LDL, copper-oxidized LDL (CuOx-LDL), and phosphocholine modified bovine serum albumin (PCho-BSA) were measured. Compared to the control group, IgM antibodies to MAA-LDL (Fig 1) increased remarkably in both the Rgp44 (p < 0.001) and *Pg* (p < 0.001) groups at the end of the study. IgM antibodies to CuOx-LDL (Fig 2) increased significantly in the mice immunized with Rgp44 (p < 0.001) and *Pg* (p < 0.001) showing no difference between the two immunizations. Substantial elevation of IgM antibodies to PCho was also found in Rgp44- (p < 0.001) and *Pg*- (p < 0.001) immunized mice when compared to controls (Fig 3).The IgM antibodies to PCho in the Rgp44 group were even significantly higher than those of the *Pg* group (p = 0.023). Plasma IgG antibody levels to MAA-LDL (Fig 1), CuOx-LDL (Fig 2), and PCho (Fig 3) did not increase and were similar in all immunization groups during the study.

**Fig 4:**
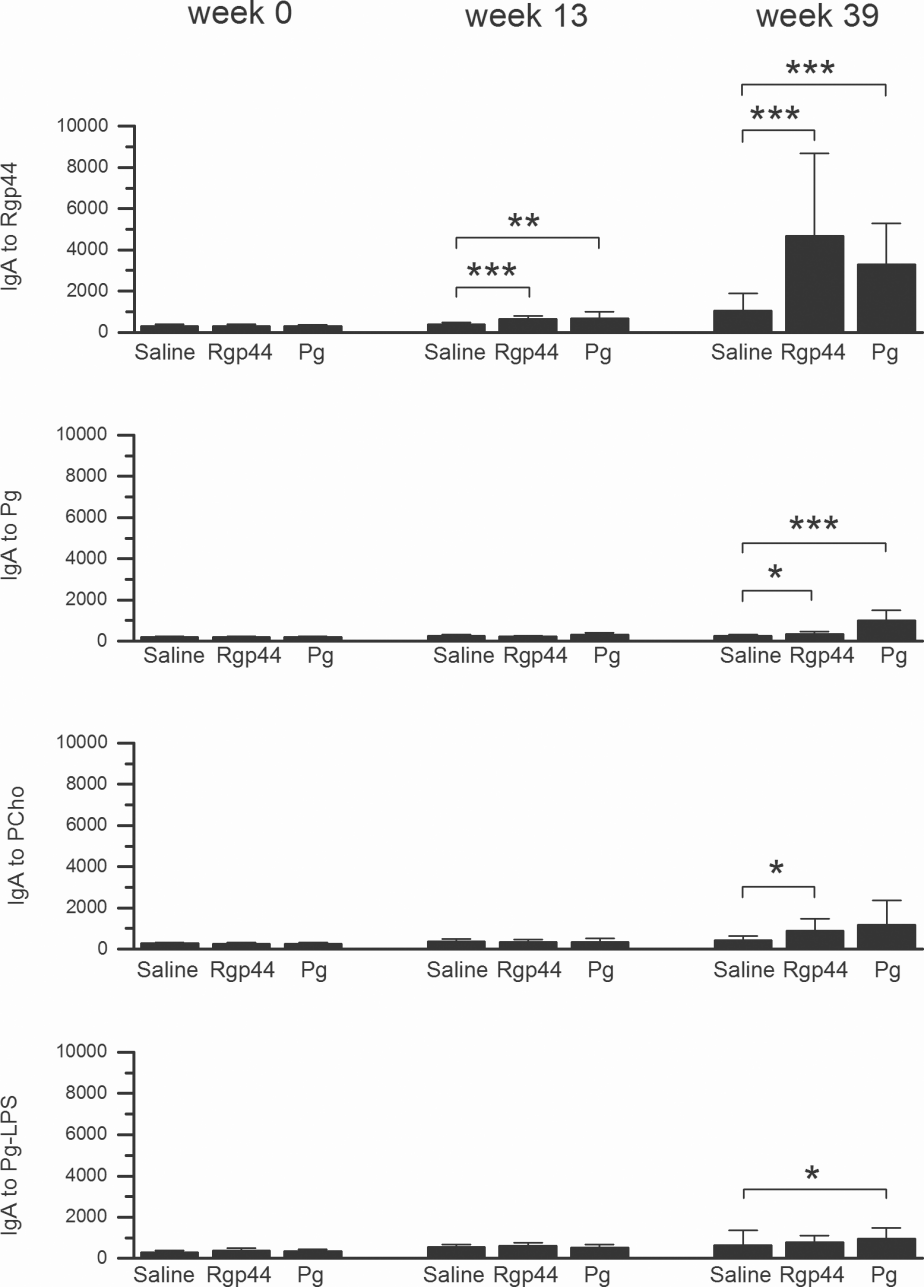
Mouse plasma IgA levels to Rgp44, *Pg*, PCho, and *Pg*-LPS before immunization (week 0), before HFD (week 13), and at the end of the study (week 39) in LDLR^-/-^ mice immunized with saline, Rgp44, and heat-inactivated *Pg*. Each mouse plasma sample was measured in triplicate using chemiluminescence immunoassay. The IgA antibody binding is expressed as mean ± SD in relative light units measured in 100 milliseconds (RLU/100ms). P-values less than 0.05 are regarded as statistically significant (nonparametric Mann-Whitney U test). * p < 0.05, ** p < 0.01, *** p < 0.001.

The IgA to OxLDL, PCho, and bacterial antigens were studied. Plasma IgA levels to MAA-LDL were significantly elevated only in the *Pg* group (Fig 1). IgA levels to CuOx-LDL remained similar in all immunization groups (Fig 2). Prominent plasma IgA responses to Rgp44 (p < 0.001 in both Rgp44 and *Pg* immunizations) and to *Pg* bacteria (p < 0.05 in Rgp44 immunization and p < 0.001 in *Pg* immunization) were observed in both Rgp44- and *Pg*-immunized mice (Fig 4). Plasma IgA antibody levels to PCho increased only in Rgp44-immunized mice at the end of the study (Fig 4). *Pg*-immunized mice, but not Rgp44 or control mice, had increased plasma IgA to *Pg* lipopolysaccharide (*Pg*-LPS) (Fig 4).

### High plasma IgA levels to Rgp44 were associated with less atherosclerotic lipid deposits in Rgp44-immunized mice

**Fig 5:**
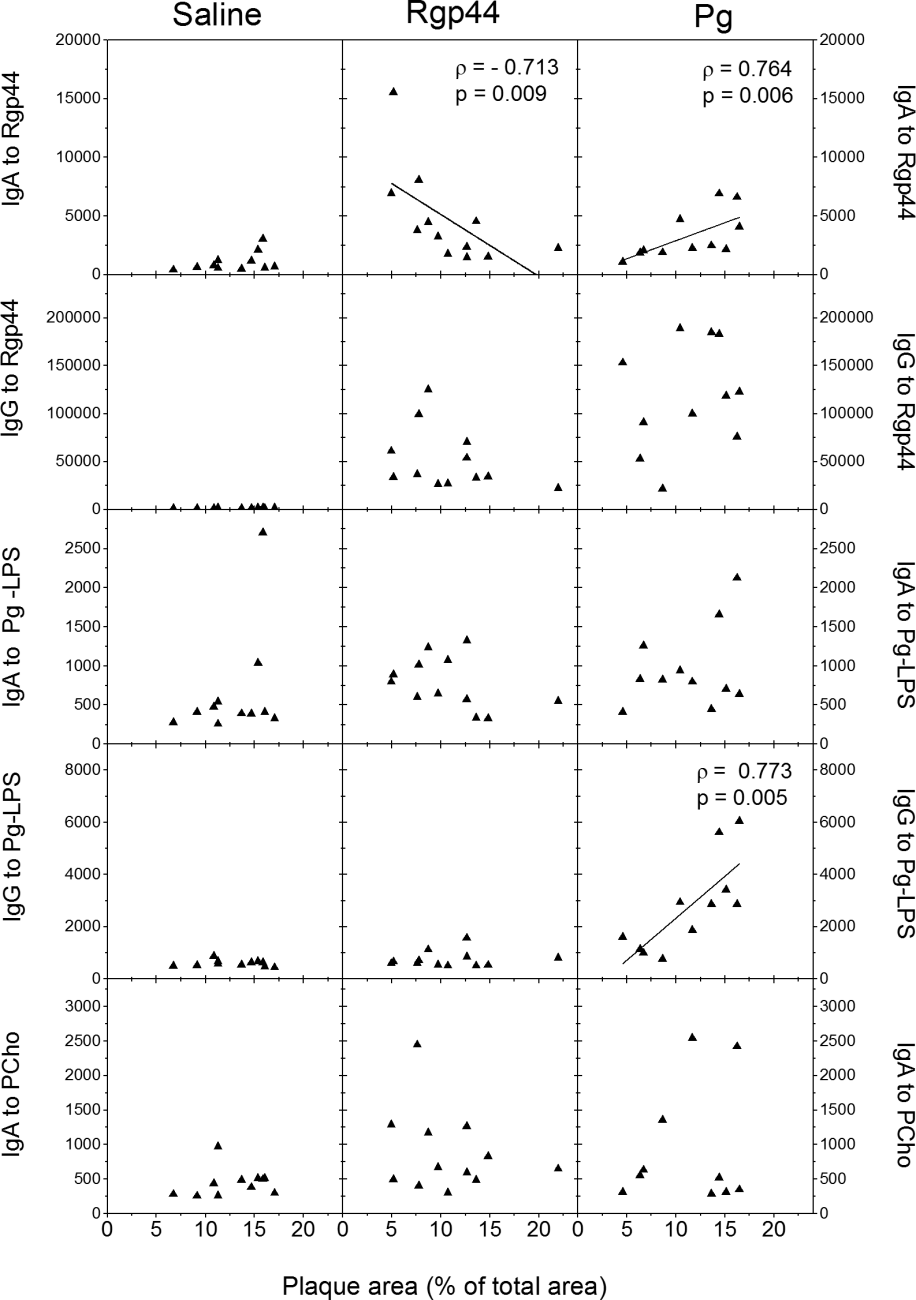
Association of mouse plasma IgA and IgG levels with atherosclerotic plaque area in LDLR^-/-^ mice immunized with saline, Rgp44, and heat-inactivated *Pg*. Plasma IgA and IgG to Rgp44 and *Pg*-LPS as well as plasma IgA to PCho were determined in triplicate by chemiluminescence immunoassay. The antibody level is expressed as relative light units measured in 100 milliseconds (RLU/100ms). Associations are determined using Spearman rank correlation coefficient (ρ). P-values (p) less than 0.05 are regarded as statistically significant.

To investigate whether the immune responses to Rgp44 or *Pg* modulated the development of atherosclerosis, atherosclerotic lipid deposition in aorta was stained with Sudan IV (S3 Fig). The stained aortic plaque areas were measured and the results were expressed as percentage of the plaque area per total area of aorta (*en face* analysis) (S3 Fig). The plaque size varied from approximately 5 to 22% of the total aortic area. The mean values of the aortic lesion area were 12.89%, 10.86%, and 11.28% in the saline-, Rgp44- and *Pg*-immunized mice, respectively. Although no significant differences were found between the three immunization groups, there was less plaque area in Rgp44- and *Pg*-immunized mice (S3 Fig). More importantly, Rgp44-immunized mice with high plasma IgA antibody levels to Rgp44 had less atherosclerotic lipid deposits compared to those with low plasma IgA to Rgp44. There was a significant negative association between the plaque area and plasma IgA levels to Rgp44 (ρ = - 0.713, p = 0.009, Fig 5). In contrast, LDLR^-/-^ mice immunized with the whole *Pg* bacteria having high plasma IgA levels to Rgp44 and high IgG levels to *Pg*-LPS had more atherosclerotic lipid deposits compared to those with low antibody levels. There was a significant positive association between the plaque area and plasma IgA levels to Rgp44 (ρ = 0.764, p = 0.006, Fig 5) and plasma IgG levels to *Pg*-LPS (ρ = 0.773, p = 0.005, Fig 5) in *Pg*-immunized mice. Plasma IgG and IgM levels to Rgp44, *Pg*-bacteria, PCho, and OxLDL were not associated with the atherosclerotic lipid deposits in any of the groups (data not shown). In addition, plasma IgA levels to *Pg*-LPS and PCho were not associated with the amount of atherosclerosis lipid deposits in any of the groups (Fig 5).

### Rgp44-immunized mice with lower plasma IL-1α levels had less atherosclerotic lesions

**Fig 6:**
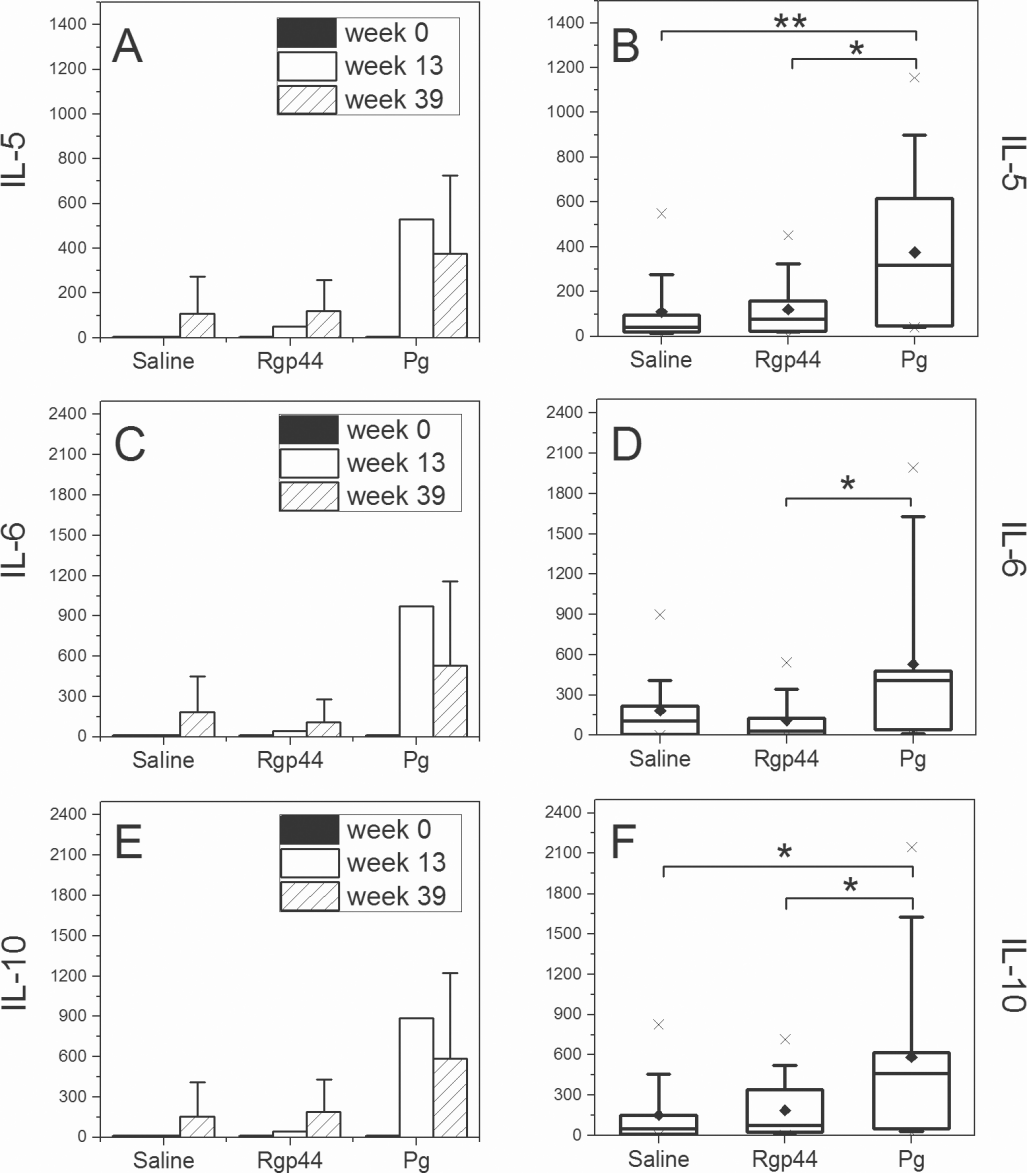
Mouse plasma IL-5, IL-6, and IL-10 cytokine levels in LDLR^-/-^ mice immunized with saline, Rgp44, and heat-inactivated *Pg*. Mouse plasma cytokine levels were determined with cytometric bead assay (CBA) from each mouse plasma sample and expressed as picograms per milliliter plasma (pg/mL). Samples at week 0 and week 13 were pooled together within each immunization group. At week 39 the plasma samples were not pooled, but measured as individuals. (A, C, E) Plasma cytokine levels at different time points within immunization groups (mean value). (B, D, F) Plasma cytokine levels at the end of the study (week 39) as box-whisker plots represent 25%, 50% and 75% of the distribution, where the whiskers represent 10% and 90% distribution of the values and the cross represents the maximum and minimum range. The solid diamonds are the mean values. * p < 0.05, ** p < 0.01.

To further investigate inflammatory responses to Rgp44 and *Pg*, plasma interleukin levels were measured. Mouse plasma IL-5, IL-6, and IL-10 levels (pg/mL) at three different time points were shown (Fig 6A, 6C and 6E). Plasma IL-5, IL-6, and IL-10 levels increased in all immunization groups. IL-5 and IL-10 levels were also significantly higher in the *Pg* group (Fig 6B and 6F) than in the Rgp44 and saline groups at the end of the study (week 39). In addition, IL-6 level in *Pg*-immunized mice was significantly higher than in Rgp44 mice (Fig 6D). No significant difference in plasma interleukin levels was observed between the Rgp44 and saline immunization groups.

**Fig 7:**
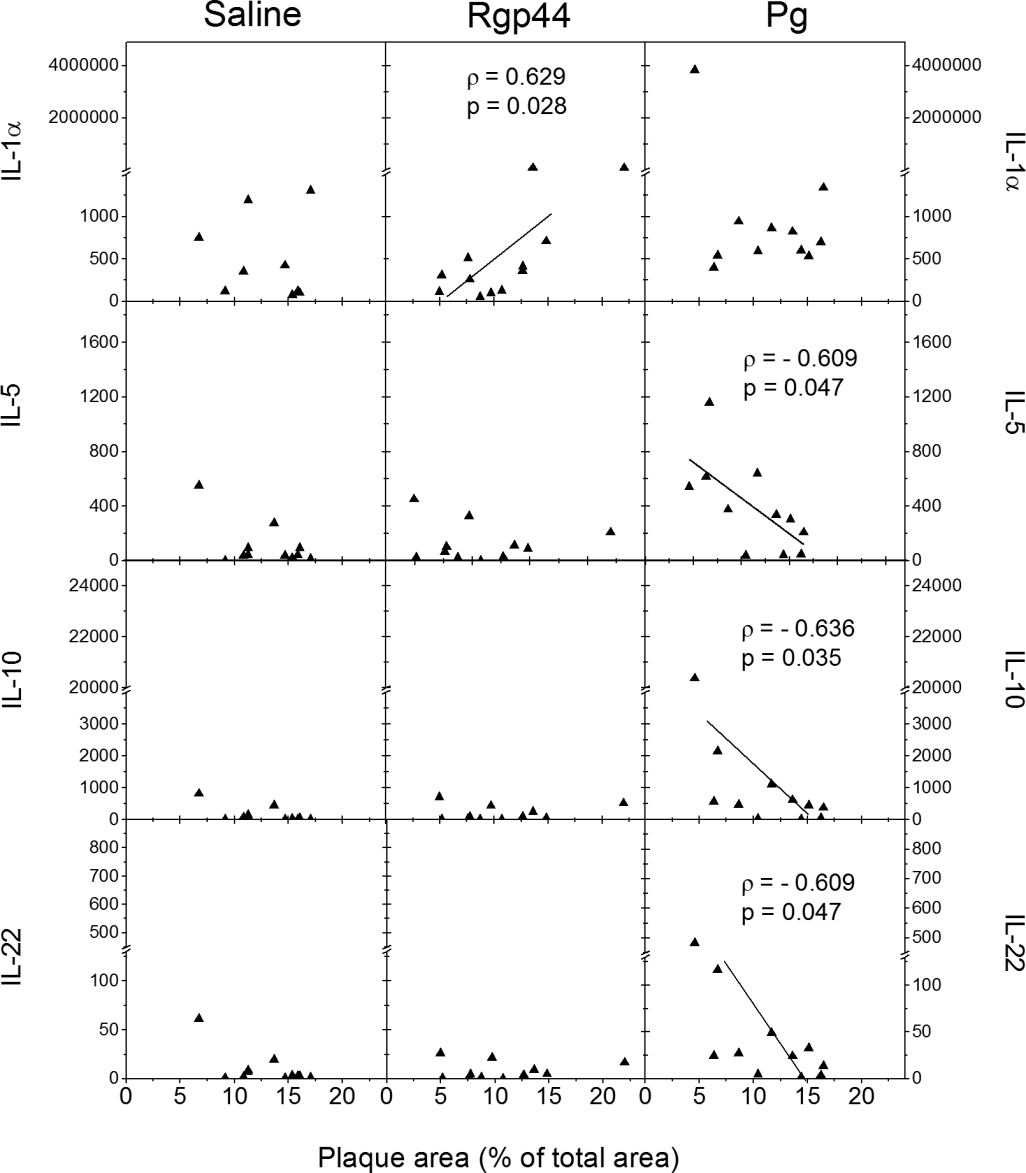
Association of mouse plasma IL-1α, IL-5, IL-10, and IL-22 cytokine levels with atherosclerotic plaque area in LDLR^-/-^ mice immunized with saline, Rgp44, and heat-inactivated *Pg*. Mouse plasma cytokine levels were determined with cytometric bead assay (CBA) from each mouse plasma sample. They are expressed as picograms per milliliter plasma (pg/mL). Associations are determined using Spearman rank correlation coefficient (ρ). P-values (p) less than 0.05 are regarded as statistically significant.

LDLR^-/-^ mice immunized with Rgp44 and with less atherosclerosis had lower IL-1α levels than those with extensive atherosclerosis. There was a significant positive association between plaque area and plasma IL-1α levels (ρ = 0.629, p = 0.028, Fig 7). The other measured plasma interleukins (IL-2, IL-5, IL-6, IL-10, IL-13, IL-21, IL-22, IL-27, INF-γ or TNF-α) were all similar in Rgp44-immunized mice compared to the saline group. None of them associated with the plaque area in Rgp44-immunized mice (Fig 7, S4 Fig). LDLR^-/-^ mice immunized with *Pg* had increased plasma IL-5, IL-10, IL-21, IL-22, INF-γ and TNF-α levels compared to the saline control (Fig 7, S4 Fig). *Pg*-immunized mice with extensive atherosclerosis had lower IL-5, IL-10 and IL-22 levels than those with less atherosclerosis. A significant negative association was observed (Fig 7) between plaque area and IL-5 (ρ = - 0.609, p = 0.047), or IL-10 (ρ = - 0.636, p = 0.035), or IL-22 (ρ = - 0.609, p = 0.047). None of the other measured interleukin levels (IL-1α, IL-2, IL-6, IL-13, IL-21, IL-27, INF-γ or TNF-α) associated with the plaque area in *Pg*-immunized mice (Fig 7, S4 Fig). Saline-immunized mice showed no association between the atherosclerotic lipid deposits and any of the plasma interleukin levels tested (Fig 7, S4 Fig).

## Discussion

The present study investigated in detail the immune responses to Rgp44, a gingipain A hemagglutinin domain of *Porphyromonas gingivalis (Pg)* which is one of the most important pathogenic bacteria in chronic periodontitis. Immunization with Rgp44 led to IgM but not IgG production against MAA-LDL, CuOx-LDL, and PCho. In other studies, these kinds of humoral changes have previously been linked to atherosclerosis. A significant negative association was observed between atherosclerotic lesion and plasma IgA to Rgp44 in Rgp44 immunized mice, indicating further an anti-atherogenic effect of the immunization.

OxLDL contains various types of immunogenic epitopes derived from modified proteins and lipids. In recent years, the MAA epitope has gained more attention because it is a closely related structure to MDA and is proposed to be one of the main epitopes after MDA modification [4–7]. MAA adduct is known to be a significant target for human natural antibodies in newborns and premature babies [5]. MAA-specific Fab antibodies have been cloned from a phage display library constructed from umbilical cord blood lymphocytes. They recognize apoptotic cells and may play a role in immunomodulation of atherogenesis [5]. Furthermore, saliva samples from healthy humans have recently been reported to contain IgA antibodies to MAA-LDL which cross-react with the periodontal pathogen *Porphyromonas gingivalis (Pg)* [25], although the autoantibody levels to OxLDL in saliva are not associated with the autoantibody levels to OxLDL in plasma. More recently, an ELISA (enzyme-linked immunosorbent assay) with purified MAA as antigen has been established, offering a specific and sensitive detection of anti-MAA antibody titer at a very early stage of atherosclerosis [6].

The role of plasma autoantibodies to OxLDL in cardiovascular disease (CVD) remains debated. Growing evidence indicates that high levels of IgM antibodies to OxLDL are inversely associated with cardiovascular disease, suggesting a protective role for IgM antibodies [5,9,18–20]. It is not yet fully understood by which mechanisms the IgM antibodies confer atheroprotection. One explanation is that they bind to OxLDL and inhibit its uptake by macrophage scavenger receptors [26]. In this study, significant elevation of plasma IgM antibody levels, but not IgG levels, to MAA-LDL, CuOx-LDL, and PCho was observed in Rgp44- and *Pg*-immunized mice. The results suggest that the induced IgM antibodies to OxLDL may attenuate the proatherogenic effect of oxidative agents, advocating atheroprotective roles for both Rgp44 and *Pg* immunizations. It has not yet been fully understood whether the molecular mimicry between the periodontal pathogens and the oxidation-specific epitopes is involved in the pathogenesis of atherosclerosis. It is widely accepted that the antibodies raised against one antigen may cross-react with another if they share sufficiently similar structures. A natural mouse monoclonal IgM antibody (Aa_Mab) cloned against MAA-LDL has been shown to recognize epitopes from chaperonin 60 (heat shock protein 60) of *Aggregatibacter actinomycetemcomitans (Aa)*, another key microbe in periodontitis [27,28]. Structural biology studies may provide more direct evidence to confirm whether *Pg* arginine-specific gingipain A adhesion/hemagglutinin domain (Rgp44) possesses a similar structure to MAA adducts. Our data suggest that Rgp44/*Pg* bacteria and MAA adducts may share cross-reactive epitopes that induce the IgM antibody production which in turn affect the progression of atherosclerosis.

Little is known about the role of IgA in atherosclerosis. IgA antibodies in cardiovascular disease have been shown to represent a sign of recent or repeated or persistent infection; however, the significance is not yet known [29–32]. High IgA antibody level to *Pg* predicts myocardial infarction and stroke independently of established CVD risk factors [29,33]. In the present study, a significant negative association between the plaque area and plasma IgA to Rgp44 was demonstrated, providing further evidence that IgA antibodies may play an important role in atherogenesis and indicating that the immunization with Rgp44 may exert a protective effect via regulation of plasma IgA antibody levels against the protease secreted by *Pg*. Plasma IgA levels to PCho were considerably elevated in Rgp44-immunized mice; however, they were not found to be associated with the size of atherosclerosis lipid deposits. In contrast to Rgp44 immunization, IgA levels to MAA-LDL and to *Pg* lipopolysaccharides (*Pg*-LPS) were increased in *Pg*-immunized mice. Likewise, in this immunization group, a positive correlation was observed between the plaque area and plasma IgA antibody levels to Rgp44, which implies that different immunogenic molecules in *Pg* may act differently from Rgp44 in regard of their role in the development of atherosclerosis. IgA titers to Rgp44 and to *Pg* in both Rgp44- and *Pg*-immunized mice were significantly higher than those of saline control, suggesting that IgA to Rgp44 could be a surrogate marker of immunization in *Pg*-immunized mice. The antibody responses of Rgp44- and *Pg*-immunized groups resemble each other closely, providing clues that the Rgp44 domain may be important for the atheroprotective effect of *Pg* immunization. Further studies are needed to determine the possible correlation between IgA antibody and atherosclerosis.

We and others have previously demonstrated that immunization with killed whole *Pg* is atheroprotective in mouse models [21,34]. In this study, LDLR^-/-^ mice immunized with Rgp44 or *Pg* did not have significantly less atherosclerosis than controls. However, the atherosclerotic plaque areas in Rgp44- and *Pg*-immunized mice, but not in control mice, were associated with several immune biomarkers, e.g. plasma IgA to Rgp44, plasma IgG to *Pg*-LPS, as well as plasma IL-1α, IL-5, and IL-10 levels. We have previously immunized LDLR^-/-^ mice with heat-inactivated *Pg* and PBS [21]. In the current study, the same mouse strain was selected, but the duration of immunization and HFD was prolonged for 5 and 8 weeks, respectively. The area of aortic plaque in the *en face* analysis of the current study was over two-fold more than in our previous study [21]. Longer HFD may influence the progression of atherosclerosis and could thus have affected the plaque formation, confounding the protective role of Rgp44 or *Pg* immunization.

Different cytokines are expressed in atherosclerotic lesions. There is a balance between pro- and anti-inflammatory cytokines and the balance is crucial for lesion development [35,36]. In this study, positive association with atherosclerotic plaque area was only seen in IL-1α among the cytokines measured in Rgp44-immunized mice. The phenomenon was not observed in saline- and *Pg*-immunized mice, revealing a different orchestration action of cytokines between Rgp44 and *Pg* immunizations. IL-1α mediates the early phases of sterile inflammation by rapidly initiating a cascade of inflammatory cytokines and chemokines, which plays a central role in the inflammation induced by apoptotic/necrotic cell death. However, the amount of circulating IL-1α is generally very low, and rarely detected even in patients with severe infections [37]. Reduced atherosclerosis has been reported in IL-1α-deficient mice [37]. Atherosclerotic lesions have long been associated with IL-1β [38]. The differential induction and distinct roles of IL-1α and IL-1β in fat-induced vascular responses and atherosclerosis have recently been highlighted [38]. Unexpectedly, the fatty acids elicit release of IL-1α but not IL-1β, revealing a selective IL-1α-persistent pathway of vascular inflammation and pathology, and suggesting a role of IL-1α in atherosclerosis. IL-5 has been suggested to play a role in protection against atherosclerosis in humans due to the stimulation of B1 cells and the production of IgM antibodies to OxLDL [39–41]. IL-10 is an anti-inflammatory cytokine mainly produced in macrophages in local atherosclerotic plaques [42]. Lack of IL-10 impacts on the development of atherosclerosis in mice [43]. IL-10 also suppresses *Pg*-driven inflammatory responses of mucosal macrophages and may be a key mediator of endotoxin tolerance [44]. IL-22, a member of the IL-10 cytokine family, has been demonstrated to play an important role in mucosal immunity in protecting bacterial infections and regulating microbiota in the gut [45]. In our study, *Pg*-immunized mice had significantly higher IL-5, IL-10, and IL-22 titers than the Rgp44- and saline-immunized mice. These cytokines also had a significant negative correlation to the area of atherosclerotic plaque in *Pg*-immunized mice, in line with our previous findings [21,22] and demonstrating an atheroprotective effect of the anti-inflammatory cytokines. However, the atherogenic properties of IL-6 are controversial. The recombinant IL-6 aggravates atherosclerosis in apoE^-/-^ mice [46] and inhibition of IL-6 trans-signaling reduces atherosclerosis [47] whereas other studies show atheroprotective properties of IL-6 [48,49]. In our study, *Pg*-immunized mice had the highest concentration of IL-6 when compared to the other immunization groups. Atheroprotective properties of IL-6 were not observed and there was no correlation between IL-6 and total plaque area in any of the immunization groups. Overall, it is intriguing that IL-1α may exert effect on progression of atherosclerosis in Rgp44-immunized mice whereas the anti-inflammatory cytokines IL-5 and IL-10 are crucial for possible atheroprotection in *Pg*-immunized mice. *Pg* immunization seems to play a more potent role in mediating and regulating cellular inflammatory responses.

Taken together, our data suggest an atheroprotective effect for Rgp44 and *Pg* immunizations. Rgp44/*Pg* and OxLDL, especially MAA adducts, may share cross-reactive epitopes which allow a prompt IgM antibody response leading to reduced risk of atherosclerosis. Both Rgp44 and *Pg* immunizations may also provide atheroprotection via modulation of pro- and anti-inflammatory cytokines levels. This immunization study promotes our understanding of the immune responses related to the development of atherosclerosis, and may also contribute to refining the current approaches aiming to regulate immune responses and inflammatory/anti-inflammatory processes in the disease.

## Supporting information

S1 Fig Experimental timeline for immunizations, blood sample collections, and diet changes. LDLR^-/-^ mice were immunized with phosphate buffered saline (Saline, n = 14), or *Porphyhromonas gingivalis* arginine-specific gingipain A adhesion/hemagglutinin domain (Rgp44, n = 13), or heat-inactivated whole *Pg* bacteria (*Pg*, n = 13). No adjuvants were used with the immunogens. Mice were fed normal chow diet for 13 weeks followed by HFD for 27 weeks. At the end of the study, the mice were sacrificed and atherosclerosis was quantified by *en face* analysis.
(TIF)

S2 Fig Mouse plasma IgG and IgM antibody levels to Rgp44 and to heat-inactivated whole *Pg* bacteria before immunization (week 0) and at the end of the study (week 39). Each mouse plasma sample was measured in triplicate using chemiluminescence immunoassay. The antibody binding is expressed as mean ± SD in relative light units measured in 100 milliseconds (RLU/100ms). Plasma antibody levels as box-whisker plots represent 25%, 50% and 75% of the distribution, where the whiskers represent 10% and 90% distribution of the values and the cross represents the maximum and minimum range. The solid diamonds are the mean values. P-values less than 0.05 are regarded as statistically significant (nonparametric Mann-Whitney U test). * p < 0.05, ** p < 0.01, *** p < 0.001.
(TIF)

S3 Fig Quantification of atherosclerosis in LDLR^-/-^ mice immunized with saline, Rgp44, and heat-inactivated *Pg*. A) The extent of atherosclerotic plaque development was determined after HFD at the end of the study (week 39) by *en face* analysis of the aortas. Sudan IV-stained aortic plaque areas were measured. Lesion sizes at the aortas are expressed as percentage of plaque area per total area of aorta. The aortic plaque areas as box-whisker plots represent 25%, 50% and 75% of the distribution, where the whiskers represent 10% and 90% distribution of the values and the cross represents the maximum and minimum range. The solid diamonds are the mean values. P-values less than 0.05 are regarded as statistically significant (nonparametric Mann-Whitney U test). ns: not significant. B) Representative pictures of the stained aortas are shown for each group.
(TIF)

S4 Fig Association of mouse plasma IL-2, IL-6, IL-13, IL-21, IL-27, INF-γ, and TNF-α cytokine levels with atherosclerotic plaque area in LDLR^-/-^ mice immunized with saline, Rgp44, and heat-inactivated *Pg*. Mouse plasma cytokine levels were determined with cytometric bead assay (CBA) from each mouse plasma sample. They are expressed as picograms per milliliter plasma (pg/mL). Associations are determined using Spearman rank correlation coefficient (ρ). P-values (p) less than 0.05 are regarded as statistically significant.
(TIF)

## Abbreviations

BSA: bovine serum albumin
CVD: cardiovascular disease
CuOx-LDL: copper-oxidized low-density lipoprotein
HFD: high-fat diet
IFN: interferon
IL: interleukin
LDL: low-density lipoprotein
LDLR^-/-^ mice: LDL receptor-deficient mice
LPS: lipopolysaccharides
MAA: malondialdehyde acetaldehyde
MDA: malondialdehyde
OxLDL: oxidized low-density lipoprotein
PBS: phosphate-buffered saline
PCho: phosphocholine; *Porphyromonas gingivalis*, *Pg*
RLU: relative light units
TNF: tumor necrosis factor
