# Supplementary figures and images for: Immunization with gingipain A hemagglutinin domain of Porphyromonas gingivalis induces IgM antibodies binding to malondialdehyde-acetaldehyde modified low-density lipoprotein
Source: PLoS One. 2018 Jan 12;13(1):e0191216. doi: 10.1371/journal.pone.0191216 (PMC5766137; doi:10.1371/journal.pone.0191216)

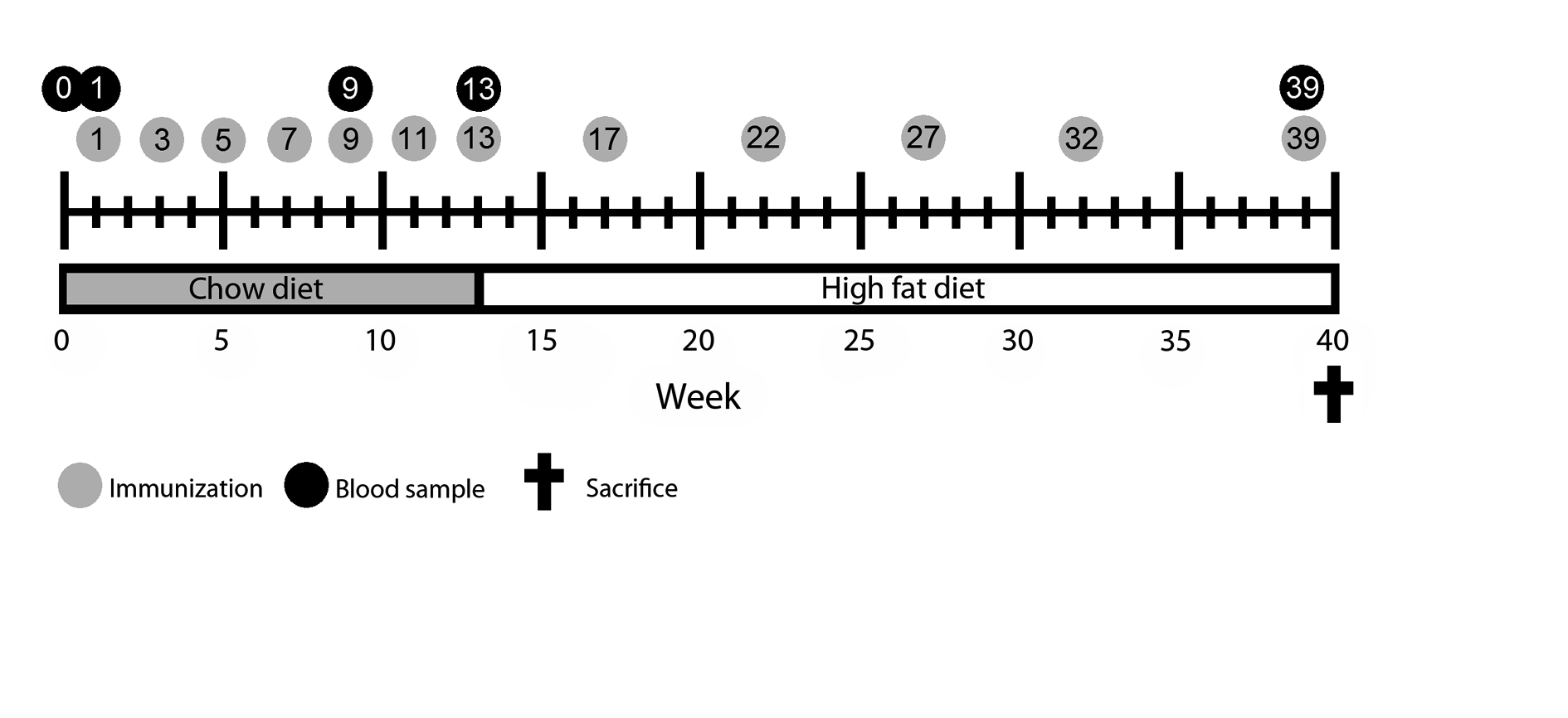

Supplement: S1 Fig — LDLR-/- mice were immunized with phosphate buffered saline (Saline, n = 14), or Porphyhromonas gingivalis arginine-specific gingipain A adhesion/hemagglutinin domain (Rgp44, n = 13), or heat-inactivated whole Pg bacteria (Pg, n = 13). No adjuvants were used with the immunogens. Mice were fed normal chow diet for 13 weeks followed by HFD for 27 weeks. At the end of the study, the mice were sacrificed and atherosclerosis was quantified by en face analysis. (TIF) [file pone.0191216.s001.tif]

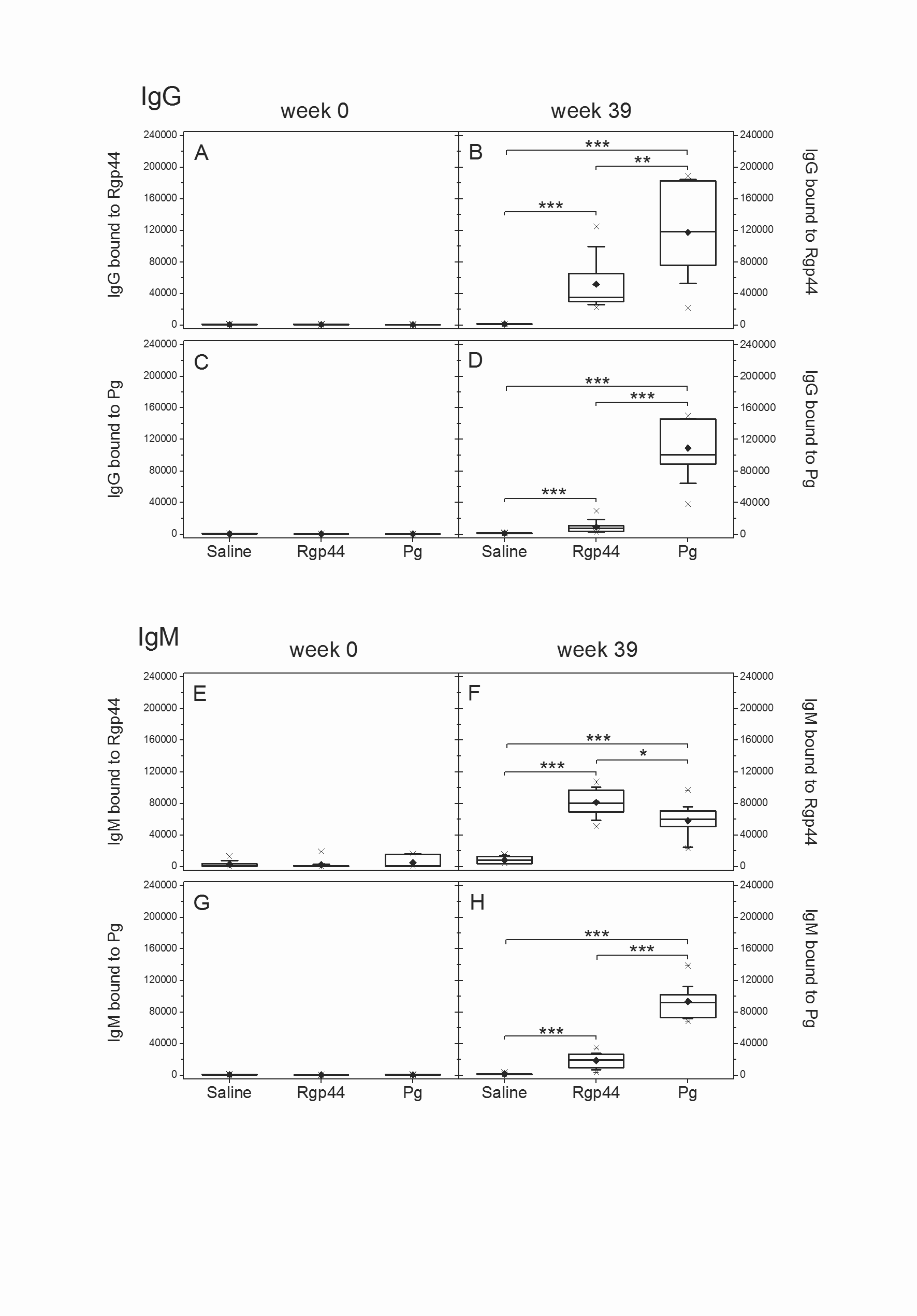

Supplement: S2 Fig — Each mouse plasma sample was measured in triplicate using chemiluminescence immunoassay. The antibody binding is expressed as mean ± SD in relative light units measured in 100 milliseconds (RLU/100ms). Plasma antibody levels as box-whisker plots represent 25%, 50% and 75% of the distribution, where the whiskers represent 10% and 90% distribution of the values and the cross represents the maximum and minimum range. The solid diamonds are the mean values. P-values less than 0.05 are regarded as statistically significant (nonparametric Mann-Whitney U test). * p < 0.05, ** p < 0.01, *** p < 0.001. (TIF) [file pone.0191216.s002.tif]

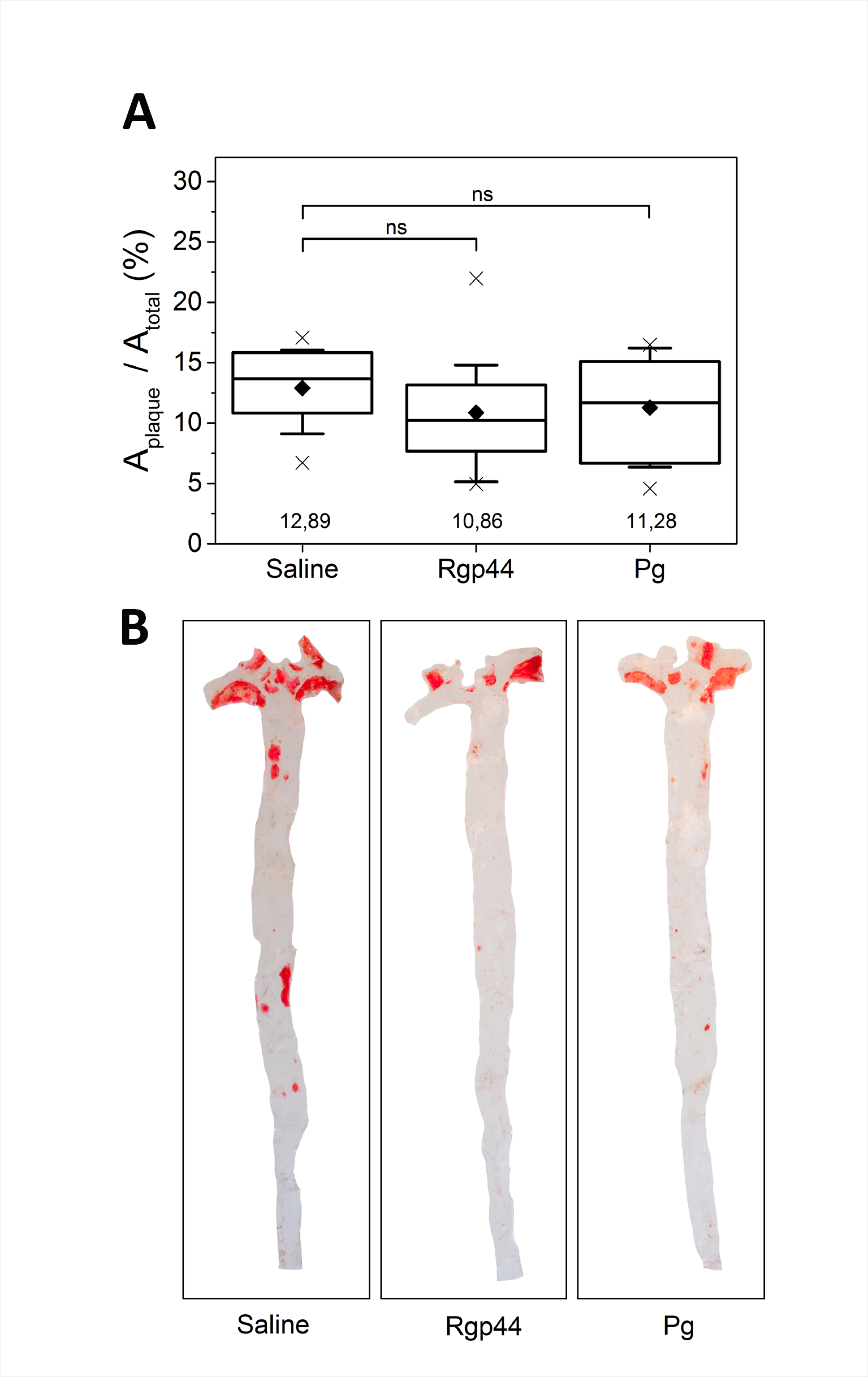

Supplement: S3 Fig — A) The extent of atherosclerotic plaque development was determined after HFD at the end of the study (week 39) by en face analysis of the aortas. Sudan IV-stained aortic plaque areas were measured. Lesion sizes at the aortas are expressed as percentage of plaque area per total area of aorta. The aortic plaque areas as box-whisker plots represent 25%, 50% and 75% of the distribution, where the whiskers represent 10% and 90% distribution of the values and the cross represents the maximum and minimum range. The solid diamonds are the mean values. P-values less than 0.05 are regarded as statistically significant (nonparametric Mann-Whitney U test). ns: not significant. B) Representative pictures of the stained aortas are shown for each group. (TIF) [file pone.0191216.s003.tif]
